# Tracing the Dynamic Chemical Transformations of Spiro‐OMeTAD in Operating Perovskite Solar Cells

**DOI:** 10.1002/advs.75107

**Published:** 2026-04-03

**Authors:** Chittaranjan Das, Mayank Kedia, Kenedy Tabah Tanko, Yunshan Wang, Christian Njel, Monica Lira‐Cantu, Michael Saliba

**Affiliations:** ^1^ Institute for Photovoltaics (Ipv) Research Center SCoPE and Integrated Quantum Science and Technology Center (IQST) University of Stuttgart Stuttgart Germany; ^2^ Helmholtz Young Investigator Group IMD‐3 Photovoltaik Jülich Germany; ^3^ Catalan Institute of Nanoscience and Nanotechnology (ICN2) CSIC and the Barcelona Institute of Science and Technology Autonomous University of Barcelona Bellaterra Spain; ^4^ Karlsruhe Nano Micro Facility KIT‐Campus North Eggenstein‐Leopoldshafen Germany

## Abstract

A significant stability challenge for perovskite solar cells (PSCs) lies in the widely used LiTFSI‐doped Spiro‐OMeTAD (Spiro) hole transport layer (HTL), in n‐i‐p configured cells. In this study, we used the sputtering depth profile X‐ray photoelectron spectroscopy (SDP‐XPS) to systematically examine the cause of instability in n‐i‐p structured PSCs under shelf‐life stability (SLS), open‐circuit potential (OCP), and maximum power point tracking (MPPT) conditions. We discover dissociation of the spiro caused by the breakdown of LiTFSI under operational stress, which alters the p‐type nature of the spiro and the interface band bending. Specifically, LiTFSI dissociation leads to the formation of byproducts, LiF and Li_X_S_Y_O_Z,_ across the HTL layer, which initiate the degradation of spiro molecules, especially under MPPT and OCP stress. While the band bending in the SLS device was around 0.6 eV, this dropped to about 0.4 eV in devices stressed by MPPT and OCP, leading to significant decreases in open‐circuit voltage and fill factor. This degradation is more severe under OCP and MPPT conditions than under SLS. By providing complex interfacial and chemical insights, this study underscores the necessity for improved dopant stability and a better HTL design to enhance the durability of PSCs.

## Graphical abstract

Mapping of Spiro‐OMeTAD layer's chemistry and electronic properties in PSC: Advanced cluster ion‐beam sputtering depth profile XPS reveals that environmental stressors (light, potential, and moisture) trigger the decomposition of LiTFSI dopants in Spiro‐OMeTAD. This chemical instability of LiTFSI degrades the Spiro‐OMeTAD layer, directly compromising the device Voc and overall long‐term stability.

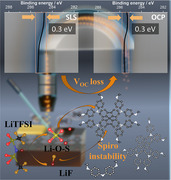

## 1 Introduction

Organic‐inorganic halide perovskite solar cells (PSCs) have evolved remarkably, reaching a certified efficiency of >27% [1, 2]. Such high performances and improved stability are mainly attributed to the advancements in additive and solvent engineering as well as interfacial passivation. The additives have been used to enhance crystallization, improve phase stability, and suppress halide segregation [3, 4, 5]. Additionally, the ubiquitous approach of interface engineering via multi‐functional organic passivation layers, laser polishing, and atomic layer deposition (ALD) has effectively suppressed interfacial defects and mitigated intrinsic halide migration [6, 7, 8, 9]. Nevertheless, the long‐term stability is hindered by the unwanted mechano‐chemical reactions at the upper interface that involve contact between the perovskite surface and the adjacent charge transport layer. This instability, caused by the degradation of the overlying charge transport layer, remains underexplored [6, 10, 11].

Currently, most high‐performance regular (n‐i‐p) PSCs are based on the benchmark hole transport layer (HTL) 2,2′,7,7′‐tetrakis[N,N‐di(4‐methoxyphenyl)amino] ‐9,9′‐spirobifluorene (Spiro‐OMeTAD, herein spiro), doped with lithium bis(trifluoromethane)sulfonimide (LiTFSI) and 4‐*tert*‐butylpyridine (tBP). The TFSI salt improves hole carrier mobility and conductivity by facilitating an in‐situ oxidation process over extended periods, while tBP enhances the solubility of dopants in spiro as well as wettability. The hygroscopic nature of LiTFSI attracts moisture, causing phase segregation on the surface and altering interface energy levels [9, 10]. Prolonged exposure to high temperatures causes tBP to evaporate from spiro, leading to an inhomogeneous distribution of LiTFSI [12]. This process created pinholes in the HTL layer, allowing moisture to interact with the perovskite and facilitating the movement of ions from the perovskite to the HTL layer. However, additional effects become more pronounced at the nanoscale level. For instance, the operating temperatures of 85°C cause spiro to transition from an amorphous to a crystalline state, inducing stress in the film and contributing to pinhole formation [13]. During operation, Li^+^ ions from the spiro and I ^−^ ions from the perovskite migrated across the HTL/absorber layer, leading to a two‐fold degradation mechanism: Li^+^ movement results in a significant loss of the photoactive properties of the perovskite, while I^−^ migration diminishes spiro, reducing the generation and separation of electron‐hole pairs [14, 15]. Current research focuses on improving the stability of spiro doped with ionic radicals, or changing the chemistry of spiro and interface with perovskite [16, 17, 18]. Additionally, alternative HTLs such as poly(3‐hexylthiophene) (P3HT) and poly(triarylamine) (PTAA) have been explored, but wettability challenges with perovskite often limit them. This limits their application to small area devices [19]. Nevertheless, to systematically investigate the overall chemical heterogeneities within the state‐of‐the‐art spiro HTL under operating conditions, a deeper understanding of fundamental reactions occurring within the spiro layer due to various dopants is required.

To study these chemical changes from the surface through the bulk of multilayer films and the buried interface, a combination of X‐ray photoelectron spectroscopy (XPS) with sputter depth profiling (SDP) is a desirable technique. This combined technique, SDP‐XPS, enables the study of the underlying degradation mechanisms in PSCs and to investigate energy level alignment under various operating conditions, which are often unreachable.

Herein, we investigated the degradation of the n‐i‐p‐based PSCs under three common operating conditions: shelf‐life stability (SLS), open‐circuit conditions (OCP), and maximum power point tracking (MPPT) using SDP‐XPS. We use a combination of monoatomic Ar^+^ SDP and cluster ion beam (C‐SDP) to study the inorganic Au layer and the organic spiro layer, respectively. At first, the depth profiles showed an inhomogeneous distribution of halide ions across various interfaces in the device under all the operating conditions. With C‐SDP, we systematically investigate the effect of LiTFSI dopant in the spiro layer under SLS, OCP, and MPPT conditions. We found that LiTSI degradation byproducts LiF and Li_X_S_Y_O_Z_ lead to the dissociation of the spiro layer, especially under OCP and MPPT conditions, resulting in a decrease in device performance. Insights into the physical and chemical dynamics at each interface enable strategies to address ion migration, material degradation, and device instability. This knowledge will guide the development of robust materials and optimized configurations, advancing stable and reliable PSC technology.

## 2 Results and Discussion

**FIGURE 1:**
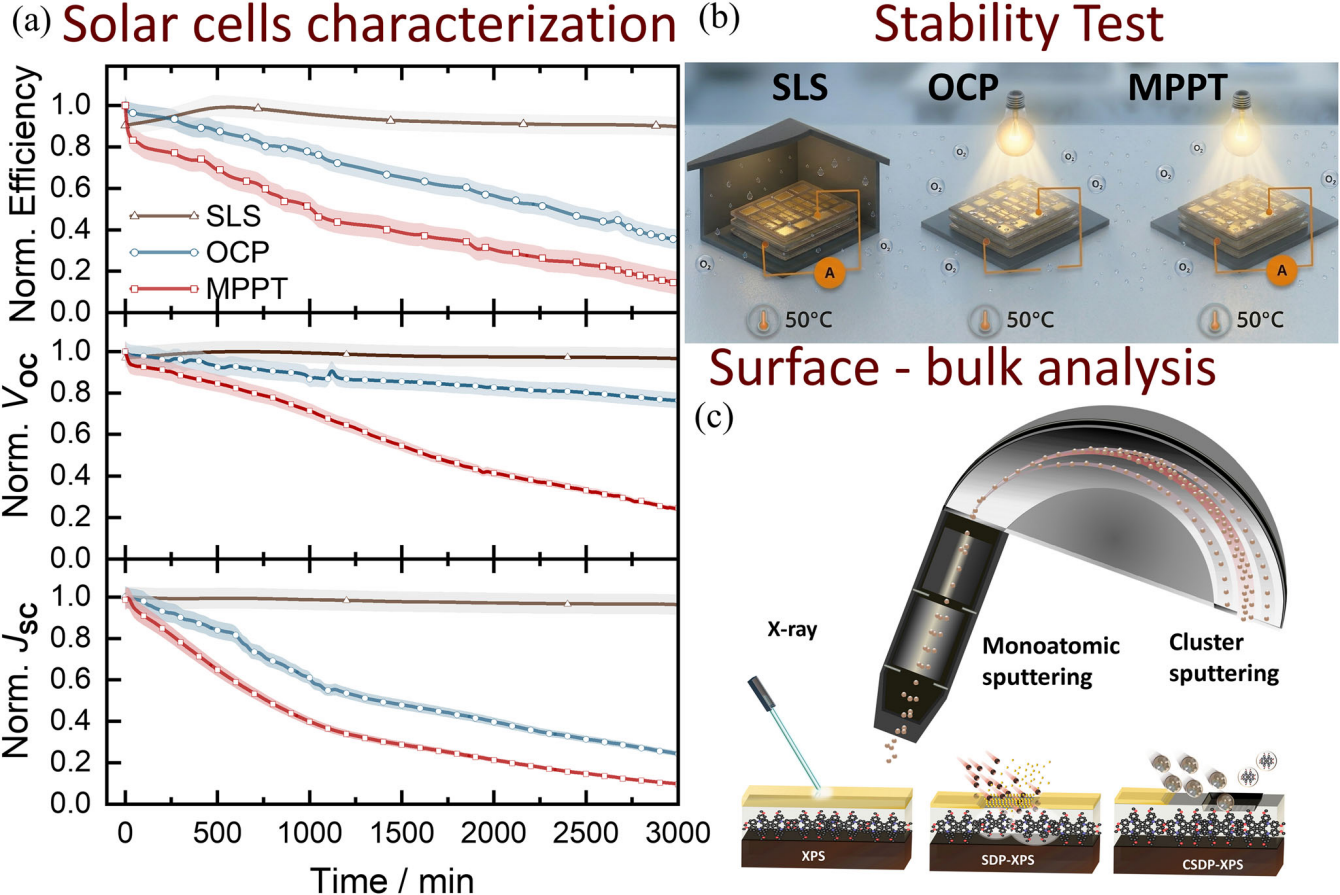
Solar cell characterization under SLS, MPPT‐ and OCP‐stressed conditions and the chemical analysis using sputtering depth profile XPS methods. Change in power conversion efficiency, Voc, and J_SC_ of solar cells at different operating conditions, the plotted data are the average from the data measured for three samples (a), and the schematic presentation of the solar cell characterization in outdoor conditions for 3000 min (b). The schematic presentation of perovskite solar cell characterization using XPS from the gold layer to the perovskite layer using sputtering depth profile methods of monoatomic and cluster ion sputtering (c).

To assess the stability of the PSCs under different stressor conditions, we examined the photovoltaic performance of n‐i‐p devices with a device stack consisting of FTO/C‐TiO_2_/M‐TiO_2_/perovskite/Spiro/Au. Here, we incorporated a triple‐cation perovskite absorber of (Cs_0.05_(MA_0.17_FA_0.83_)_0.95_Pb(I_0.83_Br_0.17_)_3_), where FA is formamidinium, and MA is methylammonium [20]. We performed current density–voltage (*J–V*) measurements on the PSCs with SLS, MPPT, and OCP at regular intervals of 20 min for 3000 min, shown in Figure 1a, and the scheme of the measurement in Figure 1b. The detailed measurement protocol is elaborated in the experimental section. The freshly prepared and SLS testing samples were stored in a dark and N_2_ environment, whereas the MPPT and OCP samples were measured in ambient air at 40% humidity without any encapsulations.

The freshly prepared PSCs have an average power conversion efficiency (PCE) of 18.5%, an open‐circuit voltage (*V*_OC_) of 1.14 V, and a short‐circuit current (*J*_SC_) of 21.5 mA.cm^−2^. A detailed *J–V* characteristic of freshly prepared cells is shown in Figure S1. When the PSC device was continuously measured under MPPT conditions for 3000 min, the PCE dropped to 35% of its initial value. Statistical data on the stability of PSCs derived from three independent samples are provided in Figure S2. Notably, a drastic decrease in device efficiency to 13% of its initial value (Figure 1a) is observed during OCP condition measurements. The reduction in device performance under various conditions is in agreement with the literature findings [21]. The underlying mechanisms for this degradation in PCE vary depending on the stress conditions. During MPPT, stress from uncollected and trapped charge carriers induces electrochemical reactions, leading to dissociation of organic cations in the perovskite and adjacent contact materials, causing a change in the optical properties of perovskite and electrical properties in the contact layers [22]. Under OCP stress conditions, the photo‐generated charges accumulate and are absorbed throughout the cell, causing a severe electrochemical reaction, halide segregation, and interfacial reactions with the contact layer [23, 24]. Quantitatively, the *J*_sc_ and *V*_oc_ of OCP‐stressed devices decreased by 90% and 75% of their initial values, respectively, while in MPPT‐stressed devices, they decreased to 75% and 23% of their initial values, respectively. The fill factor of the devices under OCP and MPPT‐stressed conditions decreased to 10% and 40% of their initial values, respectively (shown in Figure S2). The degradation of solar cell characteristics observed under both OCP and MPPT‐stress conditions is likely due to an increase in series resistance, a decrease in shunt resistance, or a combination of these two factors.

To investigate the factors contributing to the deterioration in performance of PSCs under stress conditions, we investigated the morphology, crystallinity, and optical properties of the perovskite layer within the complete device stack. The cross‐sectional Scanning Electron Microscopy (SEM) images of the solar cells in the SLS, MPPT‐stressed, and OCP‐stressed devices are shown in Figure S3. In SLS PSCs, the perovskite layer predominantly exhibits a columnar structure without any visible grain boundaries. However, upon MPPT stress, the interface with spiro deforms and becomes rough as depicted in Figure S3b. This phenomenon becomes more pronounced in the OCP‐stressed PSCs, where the interface also develops a cracked layer, as illustrated in Figure S3c. In addition, the perovskite/spiro interface became rougher from SLS to MPPT‐stress, and in the case of OCP‐stress at this interface, the perovskite layer partly detached from the spiro layer mechanically. The crystallinity of the perovskite absorber layers of the stressed solar cells is shown in Figure S4. Across all devices, the ratio between the PbI_2_ and perovskite peak intensities remains unchanged under stressed conditions. This observation suggests that the applied stress has a minimal effect on the crystallinity of the perovskite absorber within these solar cells.

The morphological (SEM) and crystallographic (XRD) studies indicate slight changes in the absorption layer of the perovskite under various stress conditions in PSC devices. This implies that the decrease in device efficiency under stress conditions largely stems from changes at the interfaces within the cells. Several interfaces are crucial for the efficient functioning of the current n‐i‐p structure of PSCs. The interface of perovskite with chemically stable TiO_2_ is considered less impactful in destabilizing the PCE of PSC devices [25]. Under stress tests, such as continuous exposure to light and biasing, interfaces at the rear side, specifically the spiro/perovskite and Au/spiro interfaces, undergo chemical changes [26, 27, 28]. The alternation in chemical composition and properties of the layers at these interfaces disrupts the smooth flow of charge carriers, ultimately impacting the performance of the PSC device. Despite minimal changes in crystallinity as well as in morphology in the stressed device, the impact on interfacial chemistry remains crucial. To investigate the interfacial chemical reactions, we employed sputtering depth profile X‐ray photoelectron spectroscopy (SDP‐XPS) to study the buried interfaces of these aged PSCs with a monoatomic Ar^+^ (1 keV) and a cluster of Ar atoms (2000 Ar gas atoms, 2 keV), as shown in Figure 1c.

**FIGURE 2:**
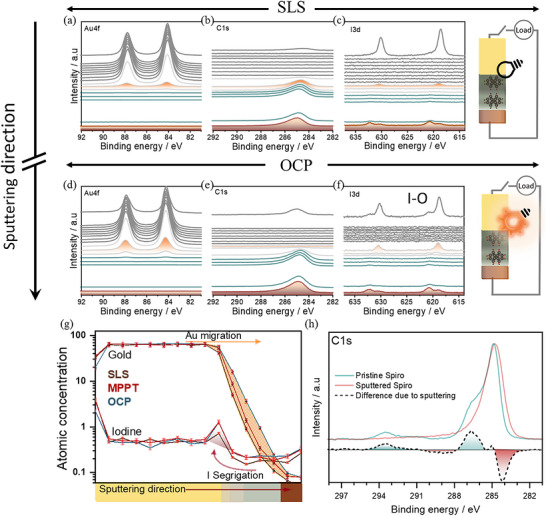
Core‐level spectra were collected from the monoatomic Ar^+^ sputtering depth profile XPS of perovskite solar cells and the elemental distribution from the Au to the perovskite layer through the spiro layer. Au4f, C1s, and I3d from the SLS (a–c) and OCP‐stressed (d–f) devices, and the distribution of Au and I from the Au layer to the perovskite with an error margin of ±5% (g). The difference between the C1s spectra of spiro before and after sputtering (h).

To begin with, we performed depth profiling of the shelf‐aged, MPPT, and OCP‐stressed devices using a monoatomic Ar^+^ beam, as shown in Figure 1c. The Au4f, C1s, and I3d core level spectra collected from the surface of the Au to the perovskite layer of shelf‐aged and OCP‐stressed devices are shown in Figure 2a–f.

The Au4f spectra in Figure 2a,d of all the samples on the surface of the Au film have binding energy (BE) at ∼84.1 eV (Au4f_9/2_) and ∼87.7 eV (Au4f_7/2_), corresponding to the metallic nature of Au [29]. With the increase in sputtering time from 0 to 480 s, the Au peaks appear to be constantly decreasing without any chemical shifts. Furthermore, between 480 and 600 s, the Au peaks completely disappeared. Similarly, the C1s peak from surface contamination can be seen for both samples in Figure 2, but with different intensities and chemical positions. This also highlights that the surfaces of the OCP‐stressed devices have a higher C‐C presence due to constant illumination under ambient atmospheric conditions. The C1s peak disappears immediately after Ar+ sputtering and reappears at 480 s. On all the stressed samples’ surfaces (0 s, without any Ar^+^ sputtering), we observed a strong I3d spectrum at ∼618.7 eV (I3d_7/2_) and ∼630.2 eV (I3d_5/2_) in Figure 2. Interestingly, for MPPT and OCP‐stressed samples, a shoulder at a higher BE of ∼2.1 eV from the main peak also appears. The presence of trace Iodine on the rear metal electrode surface could have originated from surface contamination, and the contaminated species was oxidized under the stress conditions in air for MPPT‐ and OCP–stressed devices [30]. With increasing sputtering time, the I3d peaks disappear, then reappear at 480 s and again at 680 s. These critical changes in the chemical profile of Au4f, C1s, and I3d have been used to define the corresponding etch depth of distinct layers and the two distinct interfaces present in our devices: Au/spiro and spiro/perovskite. Thus, in our case, the sputtering position of 480 s (shadowed area in Figure 2a,b) can be considered as the interface of the Au/spiro and 680 s as the spiro/perovskite interface. Additional XPS high‐resolution spectra of N1s, O1s, and F1s for all three kinds of stressed samples are shown in Figure S5.

The evolution of the atomic percentage of Au and I from the surface (sputter time 0 s) to the spiro/perovskite interface (sputter time 540–720 s) of the three stressed samples (SLS, MPPT, and OCP) is shown in Figure 2c. However, the atomic concentration of Au shows that a small amount of gold has penetrated within the spiro at the Au/spiro interface, which is clearly distinguishable (Figure 2c) for OCP‐stressed conditions. We suspect that this could stem from the degradation of spiro toward porosity under operating conditions, thus paving the pathway for the migration of the first few nanometers of the metal electrode into the spiro layer [28]. While Iodine is present on the metal electrode surface (sputter time 0 s) of all the devices, the I3d peak abruptly disappears and then starts to increase at the Au/spiro interface (sputter time 540 s), as seen in Figure 2c. With subsequent sputtering, a minor Iodine concentration can be seen within the spiro (sputter time 600 s) in Figure 2a,b. In PSCs, ions, especially iodine, migrate when the device is under non‐equilibrium conditions [31]. The SDP profile for SLS indicates that the iodine migrates to the Au/spiro interface spontaneously, even if the device experiences photons and bias for a short time. However, the presence of Iodine at the Au/spiro interface is nine times more than the Iodine concentration within the spiro layer itself for the MPPT and OCP‐stressed conditions. The higher concentration of I3d was observed at the Au/spiro interface following the sequence SLS < MPPT < OCP stressed device. Such a change in concentration is minimal but noticeable in Figure 2c. We postulate that iodine migration does not follow Fick's diffusion law, but rather the affinity of Iodine is more toward metal or metal‐based halide and not the C‐dominated spiro under operating conditions (bias and light). This result agrees with the previous studies, where Iodine appears to have accumulated at the HTL/metal interface using time‐of‐flight secondary ion mass spectrometry characterization [32]. This also implies that the concept of halide migration under biasing and light in perovskite solar cells, especially within the realm of triple‐cation perovskites, may not be as impactful as in the case of other types of perovskite combinations described in the existing literature [6]. The SDP from Au to the perovskite film reveals that the stressed conditions have minimal effects on halide and gold migration in perovskite solar cells.

To understand the interfacial reactions, we analyzed the shift in the BE and the broadening of the peaks. In the SLS device, the Au4f peak BE seems to be unchanged, while the I3d_5/2_ peak shifted to higher BE by ∼0.9 eV from the Au surface toward the spiro/perovskite interface, with a second higher BE peak only visible in the spiro layer (Figure S6). No change in Au4f position toward the spiro layer shows no visible chemical reaction between these two layers. The higher BE peak of I3d_5/2_ suggests there are chemical interactions between the spiro and the migrated iodine from the perovskite, while the constantly shifted I3d_5/2_ at lower BE could represent the band bending from perovskite to the Au layer [29, 30]. In the stressed devices (MPPT and OCP), the Au4f and I3d spectral characteristics remained the same as those of the fresh device at all interfaces (shown in Figure S6). However, the BE shift of I3d on the surface of Au and the spiro/perovskite interface is ∼0.7 eV, indicating the lower band bending caused by the MPPT and OCP stress.

At the Au/spiro and throughout the spiro layer, the C1s has a single broad peak, which is different from the pristine spiro film's C1s spectra, as shown in Figure 2h. A detailed fitting of the C1s core level forms a fresh film of spiro in comparison with the sputtered spiro, as shown in Figure S7. The higher energy monoatomic SDP with an Ar^+^ ion caused molecular fragmentation of the spiro film. We reduced the energy of monoatomic Ar+ to 500 eV to etch away the Spiro layer and obtain information on the chemical nature of the perovskite, as shown in Figure S8. However, we could not detect any appreciable difference between the samples stressed under three conditions. Therefore, to explore the role of the spiro layer and its interface with the perovskite under different stress conditions in PSCs, while minimizing experimental artifacts, we performed a less destructive cluster ion sputtering depth profile (C‐SDP) XPS.

**FIGURE 3:**
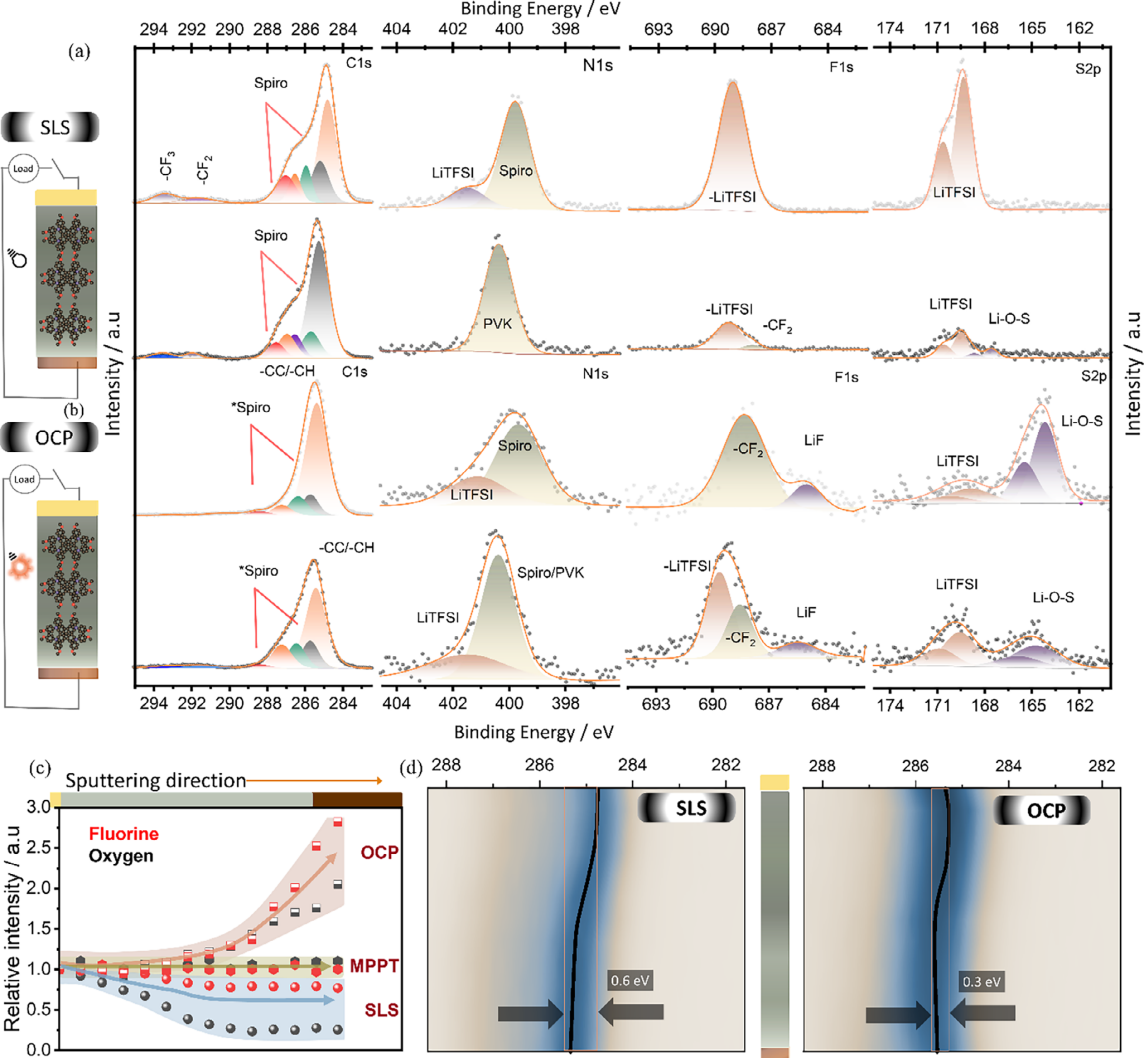
The cluster sputtered depth profile XPS study of the spiro/perovskite interface using an Ar cluster size of 2000 with 8.0 keV kinetic energy. The core level spectra of C1s, F1s, N1s, and S2p of the surface and spiro/perovskite interface for SLS (a) and OCP‐stressed (b) are shown with the elemental distribution of F and O along the spiro layer, the arrows show the trend in elemental changes (c) and the direct determination of band bending along the spiro surface to the perovskite by monitoring the C1s peak position (d).

To study the impact of C‐SDP XPS on the spiro layer from the gold/spiro interface to the spiro/perovskite interface, we focused on the elemental constituents (C1s, N1s, F1s, S2p) in the HTL layer, i.e., spiro, and the dopant Li‐TFSI. In Figure 3, the chemical nature of spiro from the surface to the interface perovskite layer in the SLS and OCP‐stressed devices is studied using C‐SDP XPS (MPPT‐stressed is shown in Figure S8).

In Figure 3a, the BE of the core‐level spectra shown in Table 1 (C1s, F1s, N1s, and S2p) at the spiro surface revealed characteristic peaks consistent with those of the LiTFSI‐doped spiro [33]. The entire depth profile spectra of all other elements are shown in Figure S8. The C1s core level spectra of the SLS device (Figure 3a) exhibited multiple sub‐peaks attributed to C‐H/‐C‐C, C─N, C─O, ─C═O, and C_a_─O bonds (aromatic carbon) within the spiro molecule, along with low‐intensity peaks at higher binding energies indicative of ─C─O─F/─CF_2_ (291.6 eV) and ─CF_3_ (293.5 eV) bonds resulting from LiTFSI interactions [31, 32]. The details B.E. and the related chemical states of these peaks have been accumulated in Table 1. At the spiro/perovskite interface, a decrease in the intensities of C1s peaks associated with specific carbon bonds (─C─C/C─H, C─N, C─O, C_a_─O, ─CF), as well as F1s and S2p peaks and in various carbon bond types, parallels the decrease in O1s intensity, suggesting a damage‐free etching. Notably, the main C1s peak (associated with ─C─H) and the N1s peak remained prominent, likely originating from the underlying perovskite layer [34, 35]. In the OCP‐stressed device (Figure 3b), the main peak of C1s has BE ∼285.3 eV, and the peaks related to C─C, C─N, C─O, and C_a_─O decreased in intensity substantially in comparison to the SLS sample (Figure 3a). In addition, the peak at a higher BE of ∼293.5 eV (─C‐F_3_) has a nominal intensity on the surface, and the intensity increases at the interface.

**TABLE 1:** Summarization of BE positions of various core levels, on the surface of the spiro layer, and at the interface.

Elements/Samples		SLS		OCP		MPPT	
		Surface	Interface	Surface	Interface	Surface	Interface
S2p	LiTFSI	169.3	169.6	169.0	169.6	168.4	169.4
	Li‐O‐S		167.5	164.3	164.8	164.5	164.9
C1s	─C─H	284.8	285.3	285.4	285.6	285.4	285.6
	─C─C	285.1	285.7	285.7	285.8	285.6	285.7
	─C─N	285.9	286.5	286.4	286.5	285.7	285.8
	─C_a_─O	286.6	287.0	287.3	287.3	286.5	286.4
	─C─O	287.1	287.6	288.4	288.4	287.2	287.2
	─CF_2_	291.6	291.8	—	291.3	288.0	288.5
	─CF_3_	293.5	293.5	293.5	293.5	289.1	290.1
N1s	Spiro	399.7	400.4	399.6	400.4	399.5	399.8
	LiTFSI			401.2	401.6	400.5	401.0
F1s	LITFSI	689.0	689.4	—	689.6	688.9	689.3
	‐CF_2_		687.7	688.4	688.4	687.4	688.0
	LiF			685.0	685.3	685.2	685.4

The F1s spectra on the surface of spiro have a single intense peak at 689.0 eV from the LITFSI dopant. At the surface with perovskite, this peak shifts to 690.4 eV, accompanied by a weak, intense peak at 687.9 eV related to the ─CF_2_ species in the SLS device. In the OCP‐stressed device, the F1s core level has a pronounced peak at 688.4 eV and a low‐intensity peak at 685.0 eV attributed to LiTFSI and LiF in the spiro layer, respectively [36]. At the interface, the peak corresponding to the –CF_3_ (689.6 eV) becomes prominent, along with a low‐intensity ─CF_2_ and LiF peak. The evolution of double peaks in the N1s spectrum at 399.6 eV and 401.2 eV on the surface and at the interface is attributed to the spiro compound and LiTFSI, respectively. The S2p spectrum peak at 169.4 eV, attributed to the LiTFSI dopant in the spiro compound, decreases sharply compared to the shelf‐aged device. Additionally, a new peak at a lower BE of 164.3 eV becomes the most prominent peak on the surface, often designated as BE for bridging S bonds in Li─S based compounds [37, 38, 39, 40]. However, the availability of a higher concentration of oxygen around the LiTFSI will cause a reaction product of the Li─O─S compound [37, 38, 39, 40, 41]. The intensities of S2p peaks at lower and higher B.E. are altered at the interface in the OCP‐stressed device. In the case of MPPT‐stress, similar patterns in C1s, F1s, N1s, and S2p were observed, as shown in Figure S9. However, the distribution of LiTFSI species of N1s at a higher BE and the S2p peak (Li─O─S) at a lower BE across the spiro layer is not as uniform as in the OCP‐stressed devices. Table 1 summarizes the BE positions and possible chemical assignments for all peaks observed in the three devices.

The BE shifts and variations in the peak intensities observed in the core‐level spectra (Figure 3) suggest potential chemical gradients within the spiro layer, extending from the surface to the interface. To quantify these observations, we calculated the atomic concentrations and relative BE changes for each element across the layer depth. Figure 3c compares the elemental distributions of F and O (C, F, O, N, and S shown in Figure S10) from the surface to the spiro/perovskite interface determined by C‐SDP XPS analysis of the C1s, F1s, O1s, N1s, and S2p core levels of the SLS, MPPT‐stressed, and OCP‐stressed devices. Notably, the SLS device exhibited higher surface concentrations of F, O, and S than at the interface, accompanied by an increase in C content. Interestingly, O decreased minutely, while N concentration remained relatively constant. This observation implies an inhomogeneous distribution of the LiTFSI dopant within the spiro layer of the shelf‐aged device. The enrichment of F and S at the surface may be attributed to their migration upon exposure to air [33]. The MPPT‐stressed device exhibits an increase in C and N content on the surface compared to the SLS device (Figure S9), whereas F, O, and S content decreased. The elemental distribution remained nearly constant throughout the spiro layer from the spiro surface to the spiro/perovskite interface (Figure S10). Conversely, in the OCP‐stressed device, there was an increase in the C content on the surface, whereas the other elements decreased relative to the SLS device. For the OCP‐stressed device, the C content decreased from the surface toward the interface, while the F and O contents increased (Figure 3c), and the N and S contents remained unchanged (Figure S10). The atomic concentration distribution of various elements within the spiro layer from the surface to the interface revealed that MPPT‐stressing induced a more homogeneous distribution of LiTFSI compared with the SLS device. In contrast, OCP‐stressing leads to the depletion of F and O from the surface of the spiro layer, which could result in changes to the chemical and electrical properties of the spiro.

To investigate potential alterations in electronic properties caused by changes in chemical composition, we monitored the shift in the BE positions of the C1s as a fingerprint of spiro (Figure 3d) for SLS and OCP–stressed samples. The BE shifts of the rest of the elements are shown in Figure S11 for the SLS, OCP‐, and MPPT‐stressed samples. In the SLS device, the BE shift of spiro‐related core levels C1s, shown in Figure 3d, by approximately 0.6 eV toward higher BE from the surface to the interface of perovskite, resembles the band bending between the HTL and perovskite [25, 26]. However, LiTFSI‐attributed core levels, such as F1s and S2p (Figure S11), exhibit a change of about 0.25 eV toward higher BE, consistent with findings in the literature [42]. In the OCP (Figure 3d) and MPPT (Figure S12)‐stressed devices, the C1s core level shifts from the surface to the perovskite interface by 0.35 and 0.42 eV toward higher BE, respectively. These shifts in the C1s BE are similar to those of the electronic shift of the undoped Spiro layer on the perovskite [43]. In the OCP‐stressed device, the BE shifts exhibit variation; N1s, S2p, O1s, and F1s core levels shift by approximately 0.45, 0.7, 0.7, and 1.2 eV, respectively. These shifts indicate a significant chemical change, resulting in the distinct electronic properties of the HTL from the surface to the perovskite interface. Under the MPPT‐stress conditions, core levels shifted by 0.42 ± 0.05 eV (C1s, N1s, O1s, and S2p) and 0.65 ± 0.05 eV (F1s) (Figure S11) toward higher BE at the interface. This change in the BE of individual spectra in both stressed conditions indicates the impact of continued operation on the band bending at the HTL/perovskite interface, likely driven by chemical changes in the HTL.

The observed reduction in the intensity of the C_a_─O, C─O, and C─N peaks within the C1s spectrum of the OCP and MPPT‐stressed device may originate from the cleavage of ─OCH_3_ from the phenyl group, ─OCH_3_, and the breaking of one or more methoxyphenyl groups from the spiro bifluorene backbone. In particular, the literature suggests that pristine spiro chemistry exhibits significant stability when exposed to O_2_, moisture, and elevated temperatures [13, 33]. In contrast, our findings reveal substantial degradation in the C1s spectra of the OCP‐ and MPPT‐stressed devices. This degradation stems from the dissociation of the ─OCH_3_ groups, a phenomenon previously observed. In our earlier work, we demonstrated that these dissociated fragments of the spiro hole transport material migrate into the perovskite layer. It is important to note that the spiro film studied here is part of a complete PSC device and includes a LiTFSI dopant. During device operation, the spiro layer is subjected to a combination of external factors, including O_2_, moisture, air, light, bias, and chemical interactions between adjacent layers. These multicomponent stressors likely induce degradation mechanisms in the spiro that differ from those observed in isolated conditions, as previously reported in the literature [13, 33]. The change in the chemistry of the spiro layer is likely accompanied by degradation in LiTFSI, which is particularly susceptible to aqueous and electrochemical environments [44, 45, 46]. Under the influence of an electric bias in the presence of moisture and air, LiTFSI can dissociate into various lithium‐containing fragments, such as LiF and Li_2_S/Li_x_S_y_O_z_ [44]. Additionally, byproducts including CO_2_ and HF, as well as intermediate phases like [CF_3_‐SO_2_‐N‐SO_2_‐CF_2_]^−^, –CF_3_, and SO_2_, may be formed as shown in the reaction mechanisms below [45, 46]. The mechanism of LiTFSI is derived by combining available literature and our experimental evidence from XPS analysis of the spiro layer [44, 45, 46].

Reaction Mechanism: Scheme 1 Reaction Mediated by the Intermediate Radical Formation.

$$LiTFSI\to TFSI^{-}+Li^{+}$$

$$TFSI^{-}+e^{-}\to^{\circ }{\left[CF_{2}SO_{2}NSO_{2}CF_{3}\right]}^{-}+F^{-}$$

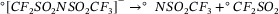




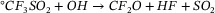

$$CF_{2}O+H_{2}O\to CO_{2}+2HF$$

$$Li^{+}{+}^{\circ }CF_{3}SO_{2}\to LiF+Li_{2}SO_{3}$$

Scheme 2 Reaction mediated by the electrochemistry route.

$$TFSI^{-}+Li^{+}+e_{ph}^{-}\to LiF+{\left[CF_{2}SO_{2}NSO_{2}CF_{3}\right]}^{-}$$

$$\begin{array}{ccc} & & 2{\left[CF_{2}SO_{2}NSO_{2}CF_{3}\right]}^{-}+e_{ph}^{-}+H_{2}O\to CO↑+\;2HF\\ & & \;+\left[NSO_{2}CF_{3}\right]+HCF_{2}SO_{2}NSO_{2}CF_{3}+SO_{2} \end{array}$$

$$Li^{+}+SO_{2}\to Li_{2}SO_{3}$$

**FIGURE 4:**
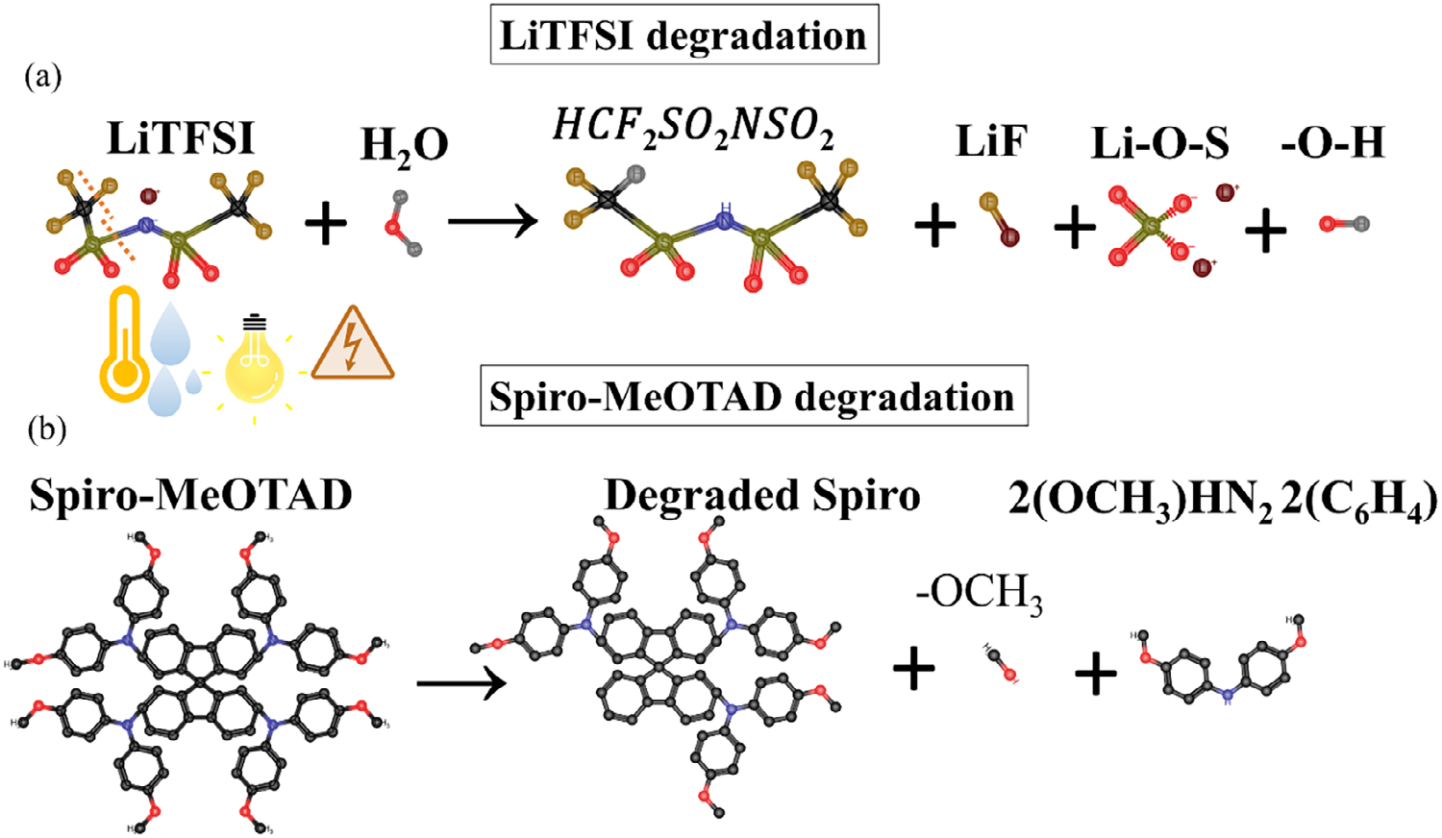
A schematic presentation of the simplified degradation process of LiTFSI (a) and the resultant spiro degradation caused by the byproduct from degraded LiTFSI (b).

The presence of peaks at 685.0 and 687.7 eV (F1s) and 164.3 eV (S2p) core‐level spectra (Figure 3b and Figure S9) corresponds to the LiF, ─CF_2_, and Li─O─S, respectively, on the surface of the stressed device. The absence of radical formation in stressed conditions and the presence of degradation products in spiro. These observations support the proposed reaction mechanism 2 for LiTFSI degradation under the influence of light, moisture, air, and favorable photo potential. A simplified schematic of the degradation of LiTFSI dopant is shown in Figure 4a.

Figure 4a illustrates a simplified degradation pathway for LiTFSI under operating conditions. In addition, the byproducts from LiTFSI, especially the unstable intermediate CF_2_O and corrosive HF, can interact with the spiro to further initiate the degradation process in the HTL film, as shown in Figure 4b. The reaction of the byproduct might have resulted in the dissociation of ─OCH_3_ from the phenyl group, the breaking of one or more methoxyphenyl groups from the spirobifluorene backbone, and the degradation of the ─OCH_3_ group, as shown in the C1s spectra in Figure 3b. In addition, the higher BE peak in C1s corresponding to the ─C─F bond also diminished from the surface, confirming the dissociation of the dopant from the spiro.

In the OPC‐stressed device, from the surface toward the bulk, the C1s spectra showed an increase in ─OCH_3_, methoxyphenyl group, and ─CF_3_, while the F1s intensity increased and the S2p peak intensity corresponding to LiTFSI also increased. In addition, the O1s and N1s (Figure S9) intensities increased from the surface of the spiro layer toward the interface with perovskite. The elemental signature and intensity distribution from the surface to the spiro/perovskite interface show that the spiro layer surface is highly degraded compared to the interface. The MPPT‐stressed device (Figure S9) also showed a similar pattern of chemical distribution from the surface to the spiro/perovskite interface. However, compared to the OCP‐stressed devices, the MPPT‐stressed devices exhibit relatively low degradation of LiTFSI as the ─CF_2_ and Li_x_S_y_O_z_ formations are lower and are uniformly distributed across the HTL layer.

The comparison between the SLS, OCP‐, and MPPT‐stressed devices shows the distinct behavior of HTL degradation, which is also reflected in the PCE under these conditions. Under the OCP and MPPT conditions, the entire device experienced a potential difference generated by incident photons or an applied bias between the anode and cathode in the ambient environment, respectively. Under these circumstances, the voltage is experienced on the cathode side, specifically at the Au/Spiro/perovskite interface in the present case, which may lead to photoelectrochemical reactions at these interfaces. In particular, the Au and TCO anodes experienced a potential of approximately 1.1 volts in the OCP‐stressed device. This resemblance to the solid electrolyte nature of the LiTFSI dopant in the HTL film could potentially cause the dissociation of LiTFSI and result in the reduction and de‐doping of the spiro layer [47]. In the MPPT‐stressed device, a voltage near the maximum output power is applied to extract the photo‐generated charges, causing the redistribution of LiTFSI and later initiating the dissociation of dopant and degradation of spiro in the HTL film.

**FIGURE 5:**
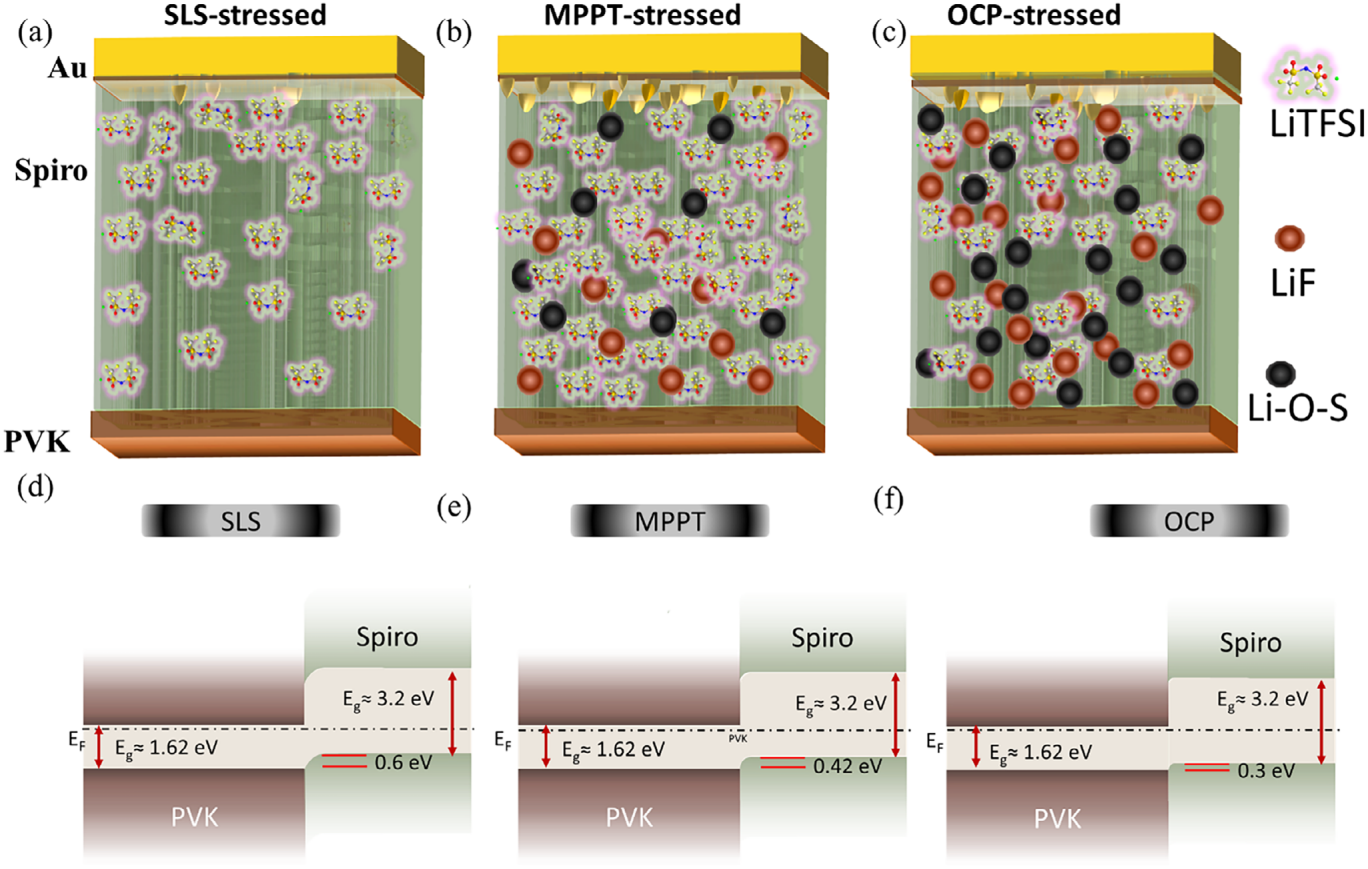
Schematic of the chemical distribution in the spiro layer and its electronic interface with perovskite and Au under different stress conditions. Figure a–c show the distribution of chemical components of spiro, from Au to the perovskite interface. The determined band edge position and the band bending at the perovskite spiro interface under stressed conditions of SLS, OCP, and MPPT are shown in Figure d–f, respectively.

The SDP XPS analysis of SLS, OCP, and MPPT stressed devices reveals how chemical changes occur within the spiro layer and its interaction with Au and perovskite at the interfaces, as well as how halide migration from the perovskite toward the metal contact impacts the overall device performance. In Figure 5, we discuss the effect of all the processes involved in determining the long‐term operational stability of the PSCs based on the chemical analysis of the spiro layer.

Our investigation of the halide migration patterns using SDP XPS analysis revealed unexpected results. We found that halide migration patterns, particularly for iodine, were consistent across the three distinct types of conditioned cells examined. Contrary to prevailing assumptions, our findings indicate a pronounced accumulation of iodine at the interface between the Au and spiro layers. In contrast, the concentration within the individual Au and spiro layers remains marginal, as depicted in Figure 5. Notably, devices subjected to MPPT‐ and OCP ‐stress exhibited an iodine concentration at the Au/spiro boundary, which was approximately 20% greater than that of the SLS devices (shown in Figure 5a–c). Despite this, the impact of iodine migration on the photovoltaic efficiency of the PSCs appears to be minimal, given the wide bandgap and triple cation composition of the perovskite employed [20]. Nevertheless, the slight migration of iodine cannot be dismissed as inconsequential, as it may influence the electronic properties of the spiro layer.

The C‐SDP XPS showed that the spiro layer underwent drastic changes under operational conditions. The LiTFSI dopant endured chemical degradation, leading to reduced doping effectiveness within the spiro layer. Furthermore, the degraded LiTFSI product compromises the chemical stability of the spiro molecule, causing its dissociation. These chemical alterations in the spiro layer contribute to several issues in PSCs, including impaired hole transport properties, physical detachment of spiro at the HTL/perovskite interface, and migration of degraded byproducts that affect the perovskite absorber layer over time. Under MPPT‐ and OCP‐ stress conditions, the formation of Li─O─S and LiF (as depicted in Figure 5b,c) occurs throughout the entire spiro thickness. However, the LiF and Li─O─S species were distributed uniformly in the OCP‐stressed device, while in the MPPT‐stressed device, the Li─O─S was more at the interface between the Au/spiro and decreased toward the perovskite interface, keeping the LiF uniform across the spiro layer (see Figure S9). The degradation of LITFSI additives reduces the p‐type doping nature of spiro, causing a decrease in band bending, reducing the band from 0.6 eV in the SLS device to ∼0.42 eV in MPPT‐ and ∼0.3 eV OPC‐stressed devices at the HTL/perovskite interface (Figure 5d,e). The change in electronic interface led to a decrease in the Voc of the device and, consequently, other photovoltaic properties, including fill factor and power conversion efficiency. The XPS studies at different interfaces reveal the nature of the chemical distribution and related changes in electronic properties of Au, Spiro layers in SLS‐, MPPT‐, and OPC‐stressed devices. Based on our findings, future efforts to enhance n‐i‐p‐type PSC device longevity should focus on replacing LiTFSI with stable, non‐lithium alternatives or employing interface modification strategies [8, 48, 49, 50]. These approaches are essential to prevent the harmful interaction between HTL degradation byproducts and the perovskite layer, thereby maintaining high performance over an extended period.

## 3 Summary and Conclusion

We investigated the operational stability of classical n‐i‐p‐structured perovskite solar cells under various operating conditions, primarily including SLS, MPPT, and OCP conditions. Despite similar changes in the perovskite bulk revealed via XRD, the rate of performance deterioration under various conditions is different. In this work, we utilized sputter depth profile XPS to assess the changes in the organic overlayer, spiro, as a possible contributor to the total device performance. We confirmed the presence of migrated Iodine ions at the Au/spiro interface in SLS devices as well as stressed devices. Further, using a cluster‐based SDP XPS profile, we observed a variation in the distribution of LiTFSI additive within the spiro layer, surface majority in the SLS device, as compared to an even distribution in the MPPT. This distribution of LiTFSI salt under various bias conditions resulted in the degradation of the spiro molecule as a whole. Under stress conditions, LiTFSI undergoes an electrochemical reaction, forming LiF and Li_x_S_y_O_z_ within the spiro. Subsequently, these byproducts initiate the dissociation of the spiro molecule into possible ─OCH_3_ and ‐2OCH_3_HN_2_ (C_6_H_4_) components, resulting in degraded HTL and an unfavorable interface.

Apart from the chemical changes, the SDP XPS reveals a total band bending across the Au/HTL/perovskite interfaces of about ∼0.9 eV, which describes the maximum obtainable photovoltage across this interface. With the use of less destructive C‐SDP‐XPS, we found at the HTL/perovskite interface that the band bending is about ∼0.6 eV for the SLS, while in the case of MPPT and OCP‐stressed, it is about ∼0.42 and ∼0.3 eV, respectively. This reduction in band bending is a direct consequence of the altered p‐type nature of the HTL, a property derived from both spiro and LiTFSI. These molecular changes in the HTL, as a whole, result in minimized *V*_oc_ and FF in the final device performance. The findings underscore the role of LiTFSI‐doped spiro‐based HTL in the overall stability of the perovskite solar cell devices.

## Conflicts of Interest

The authors declare no conflicts of interest.

## Supporting information




**Supporting File**: advs75107‐sup‐0001‐SuppMat.docx.

## Data Availability

The data that support the findings of this study are available on request from the corresponding author. The data are not publicly available due to privacy or ethical restrictions.
