# Supplementary material for: Tracing the Dynamic Chemical Transformations of Spiro‐OMeTAD in Operating Perovskite Solar Cells
Source: Adv Sci (Weinh). 2026 Apr 3;13(34):e75107. doi: 10.1002/advs.75107 (PMC13285165; doi:10.1002/advs.75107)
Supplement: Supplementary file 1 — Supporting File: advs75107‐sup‐0001‐SuppMat.docx. [file ADVS-13-e75107-s001.docx]

## Tracing the Dynamic Chemical Transformations of Spiro-OMeTAD in Operating Perovskite Solar Cells

Chittaranjan Das ^1,2†*^, Mayank Kedia ^1,2†^, Kenedy Tabah Tanko ^3^, Yunshan Wang ^1^, Christian Njel ^4^, Monica Lira-Cantu ^3^, and Michael Saliba ^1,2 *^

[a] Dr. C. Das, M. Kedia, Yunshan Wang, Prof. M. Saliba

Institute for Photovoltaics (ipv), Research Center SCoPE, and Integrated Quantum Science and Technology Center (IQST), University of Stuttgart, Pfaffenwaldring 47, 70569 Stuttgart, Germany

^*^E-mail: [chittaranjan.das@ipv.uni-stuttgart.de](mailto:chittaranjan.das@ipv.uni-stuttgart.de), [michael.saliba@ipv.uni-stuttgart.de](mailto:michael.saliba@ipv.uni-stuttgart.de)

[b]Dr. C. Das, M. Kedia, Prof. M. Saliba

Helmholtz Young Investigator Group, IMD-3 Photovoltaik, Forschungszentrum Jülich, 52425 Jülich, Germany

[c] Dr. K. T. Tanko, Prof. M. Lira-Cantu

Catalan Institute of Nanoscience and Nanotechnology (ICN2), CSIC and The Barcelona Institute of Science and Technology, Autonomous University of Barcelona, 08193, Bellaterra, Spain

[d] Dr. C. Njel

Karlsruhe Nano Micro Facility, KIT-Campus North, Hermann-von-Helmholtz-Platz 1, 76344 Eggenstein-Leopoldshafen, Germany

^†^Chittaranjan Das and Mayank Kedia are equal contributing authors.

**Experiment:**

We studied the n-i-p structured solar cells without any surface and interface modifications. The solar cell structure is consisting of FTO/C-TiO_2_/M-TiO_2_/Perovskite/Spiro/Au. After the device fabrication, initial solar cell characterization and stability test were done at the home institute (ipv). For chemical analysis of the stressed cells, they were sent to KIT for XPS experiments.

**Device Fabrication:**

The solar cell device fabrication began with the first step of cleaning the pre-patterned FTO substrates. They were washed with 2% Hellmanex detergent solution and subsequently cleaned in the ultrasonicater with water, acetone, and isopropanol for 15 min for each solution. Then, a 30 nm TiO_2_ compact layer was deposited on cleaned FTO substrates using spray pyrolysis at 450 °C with a titanium diisopropoxide bis(acetylacetonate) in anhydrous ethanol precursor. The substrates were held at 450 °C for an additional 45 minutes and then cooled. Next, a 150-200 nm mesoporous TiO_2_ layer was deposited via spin coating (10 s at 4000 rpm with 1000 rpm s^-1^acceleration) using a diluted 30 nm particle paste. The resulting substrates were heated at 100 °C for 10 minutes and finally annealed at 450 °C for 30 minutes under a dry air flow. Thereafter, the samples were treated with UV ozone for 20 min to remove organic matter and activate the surface with –OH. The substrates were then transferred to a glove box for deposition of the perovskite film. We used the CsMAFA triple-cation, Cs_0.05_(MA_0.17_FA_0.83_)_0.95_Pb(I_0.83_Br_0.17_)_3_ solution for the deposition of the perovskite thin film. The details of the perovskite solution preparation are explained somewhere else.^18^ In short, the perovskite solution was prepared by mixing FAI (1.16 M), MABr (0.24 M), PbI_2_ (1.19 M), and PbBr_2_ (0.24 M) in a solvent mixture of DMF and DMSO in a 4:1 volume ratio. Then a solution of 1.5M CsI is added to the prepared solution to obtain Cs_0.05_(MA_0.17_FA_0.83_)_0.95_Pb(I_0.83_Br_0.17_)_3._ For perovskite film preparation, the solution was spin coated on the TiO_2_ surface at 1000 rpm for 10 s and 5000 rpm for 20 s, followed by 150 µl of chlorobenzene as antisolvent. For drying and crystallization, the perovskite films are annealed at 100°C for 30 min. After cooling, the spiro-OMeTAD solution was spin coated for 20 s at 4000 rpm and allowed to oxidize for 24 hours in the oxygen-filled box. To complete the device structure, 100 nm of Au layer was deposited by thermal evaporation.

***JV* measurements**

The *J–V* characteristics of the solar cells were recorded in an ambient atmosphere under an AM 1.5 G, 100 mW cm^−2^ spectrum (WAVELABS, SINUS-70) using a Keithley 2400 source meter. The solar cells were unencapsulated during the test and measured in both forward and reverse directions using a 0.089 cm^2^ mask from −0.2 V to 1.2 V with 200 mV s^−1^.

**Stability Test**

The stability assessment of the solar cells was done in the ambient conditions without any encapsulation. For shelf life stability, the samples are stored in the dark and under an N₂ environment, and solar cell characteristics are measured at continuous intervals. For MPPT, the samples were allowed to work at the maximum power, and continuous *J*sc, *V*oc, and FF were measured. While for the OCP measurements, the samples were illuminated at equal intervals, all the solar cell characteristic measurements were done. All the solar cell characteristics were recorded in an ambient atmosphere under an AM 1.5 G, 100 mW cm^−2^ spectrum (WAVELABS, SINUS-70) using a Keithley 2400 source meter.

**Surface and Interface Characterization:**

For surface and interface chemical studies of different layers in the perovskite solar cells after the stressing test, a combination of various XPS methods, such as standard and sputtering depth profile XPS, using the Thermo Fisher Scientific K-Alpha^+^ XPS-Spectrometer equipped with a Mono Atomic and Gas Cluster Ion Source (MAGCIS) system. After the outdoor stability test, the samples were brought to KIT for surface analysis at the KMNFi facility. A monochromatic Al K-alpha source with photon energy of 1486.5 eV and an illumination area of 450 µm was used to excite the sample for photoemission of electrons from the specimen. For the Au/Spiro interface studies, we used the high etching rate monoatomic Ar ion sputtering with 1 keV of kinetic energy. To further study the Spiro/perovskite interface, we used the slow etching rate and less destructive sputtering of gas cluster beam sputtering XPS (we termed it as C-SDP XPS) using a 2000 cluster size of Ar atoms with a kinetic energy of 8000 eV. The SDP XPS were measured by 60 seconds of etching, followed by 5 seconds of waiting, and then spectral collection using Avantage software from Thermo Fisher. The core level spectra are also analyzed using the Avantage software. Avantage's inbuilt smart background is used for background correction, and peaks are fitted with a Lorentzian-to-Gauss ratio of 30%.


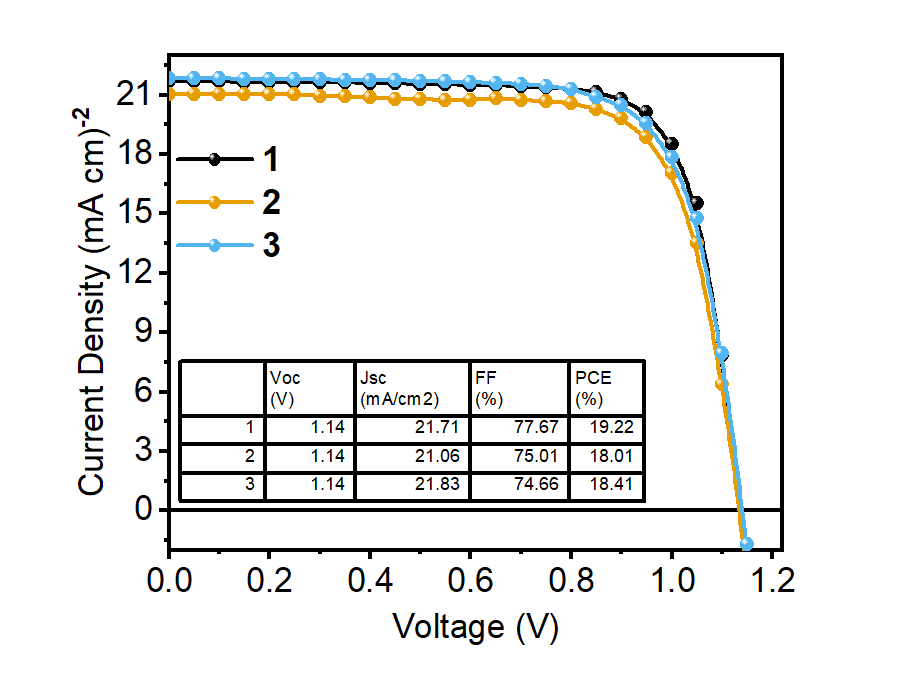


Figure S1: JV characteristics of the freshly prepared devices before being sent for stress test.


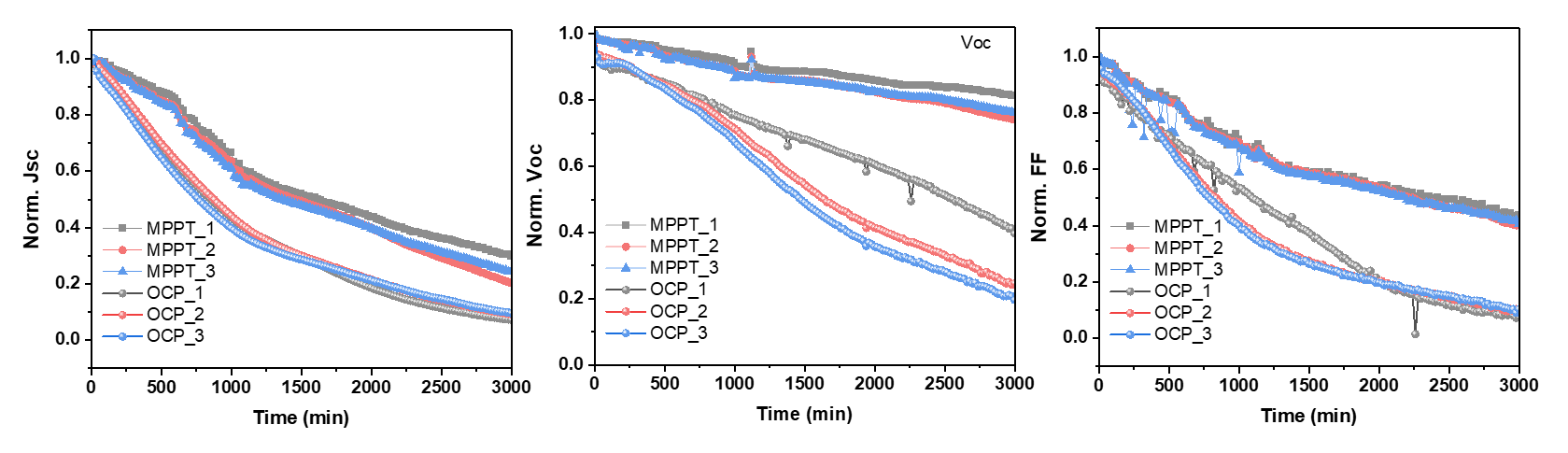


S2: The change in the efficiency of the solar cells under different operating conditions of OCP and MPPT in the outdoor facility test.


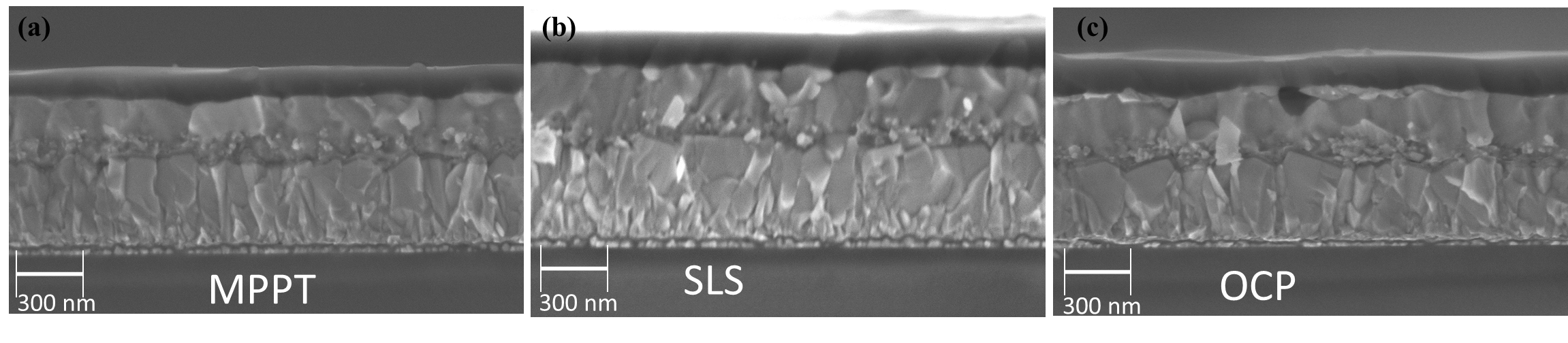


Figure S3: The cross-sectional SEM of perovskite solar cells at different stressed conditions. In the SLS and MPPT sample, the perovskite film and the spiro layer appear intact, whereas after OCP-tressing, the perovskite layer appears to break into smaller grains at the interface with Spiro.

Figure S4: The X-ray diffraction pattern of the SLS, OCP, and MPPT-stressed solar cells. The stressed devices show a significant increment in the formation of PbI_2_ in the matrix of the perovskite film in the solar cells. The relative intensity of PbI_2_ to the perovskite is slightly higher for the MPPT-stressed devices.


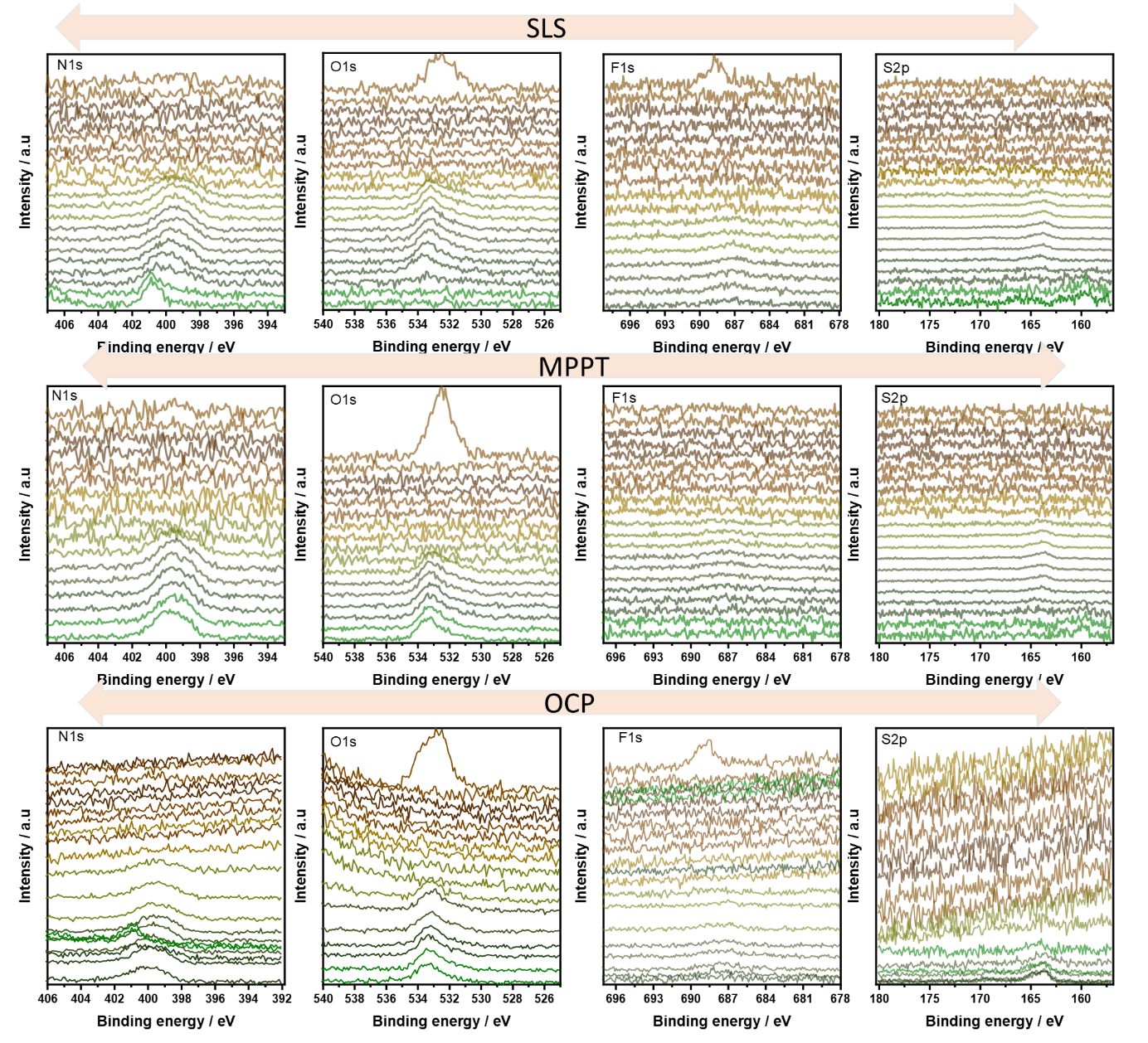


Figure 5. Monoatomic depth profile from Au to spiro layer showing the core levels N1s, O1s, and F1s from the SLS, MPPT, and OCP stressed devices. The spectra presented here, from top to bottom, are the direction of sputtering.


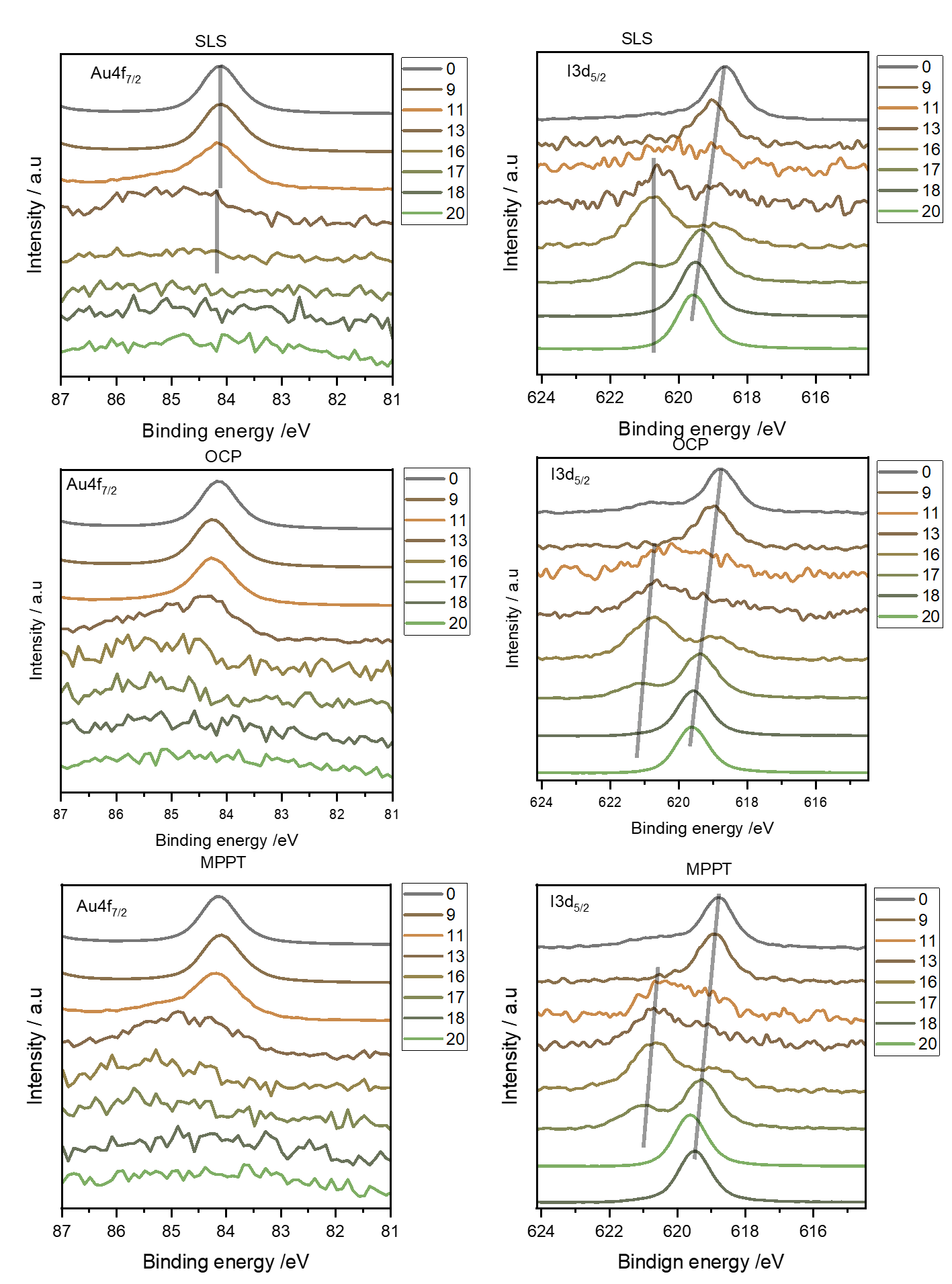


Figure S6. Monoatomic depth profile from Au to spiro layer showing Au4f_7/2_ and I3d_5/2_ spectra for SLS, OCP, and MPPT stressed devices.


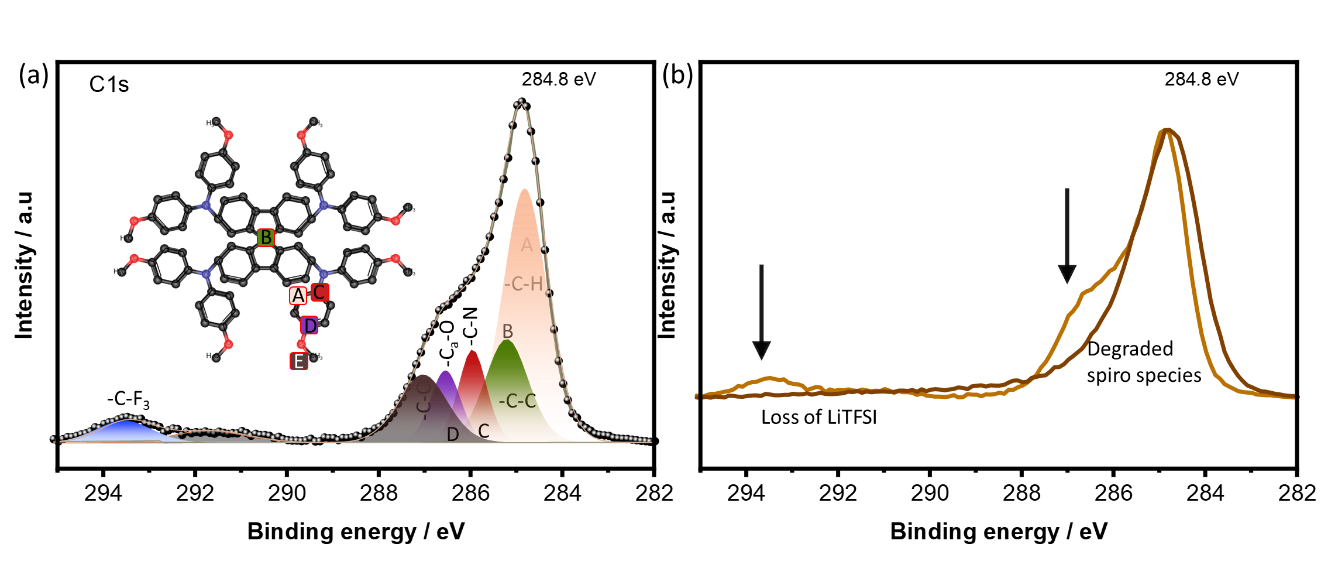


Figure S7: The C1s core level of the fresh film and the fitted with different components (a), and the comparison of the fresh and monoatomic sputter etched C1s spectra from the spiro film.


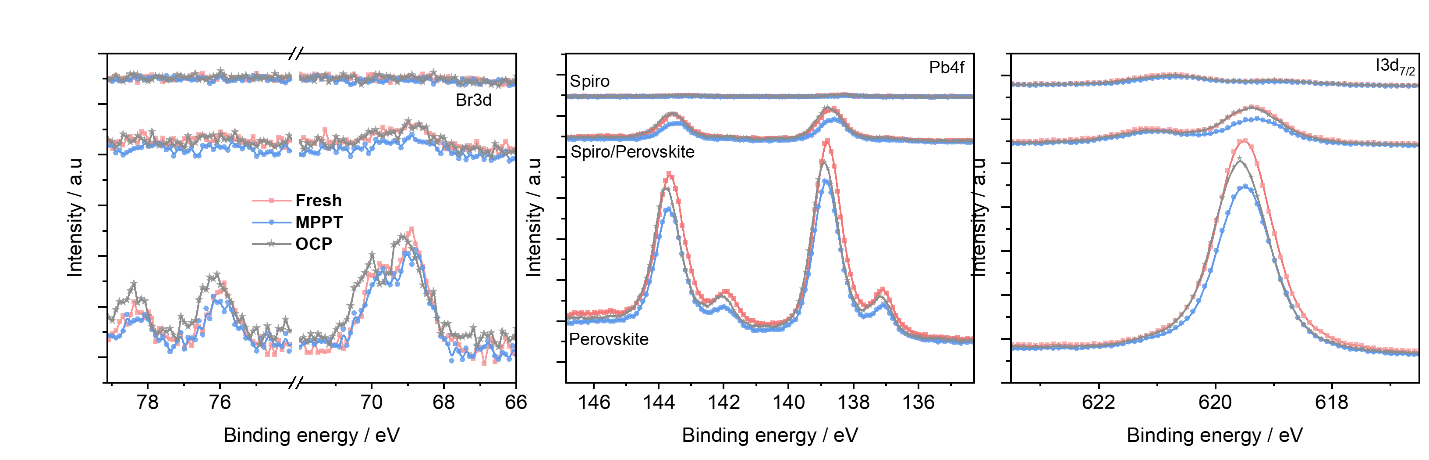


Figure S8: The interface chemistry of perovskite with Spiro after different stressing conditions. The core level spectra of Br3d, Pb4f, and I3d_7/2_ of the sputter depth profile from the spiro to the perovskite. The top spectrum is on the Spiro layer just after the etching of a thick Spiro layer with a certain amount of remnant, and the other two spectra are after the complete removal of Spiro.


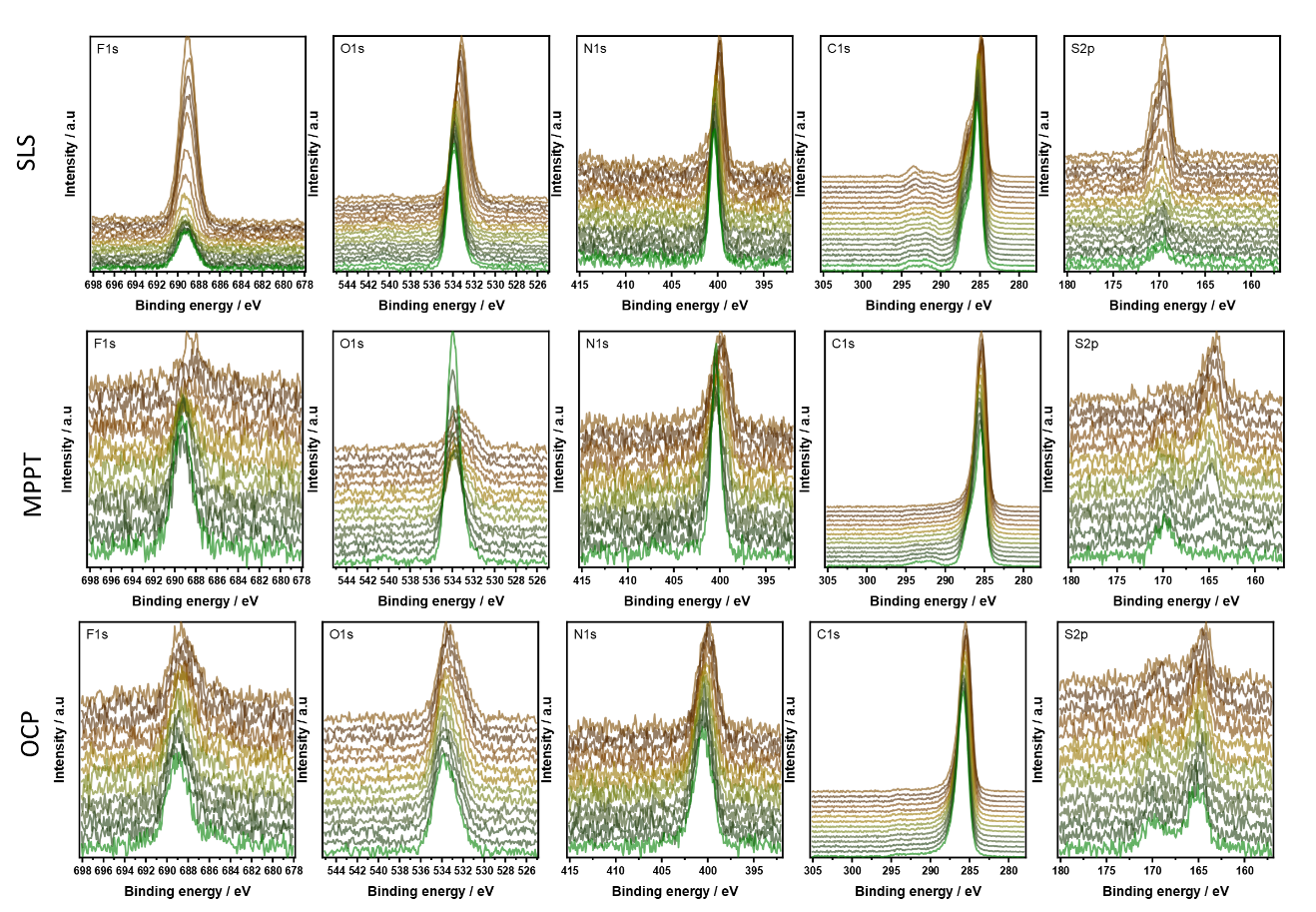


Figure S9. C-SDP XPS of all three samples (self-aged, SPO, and OCP-stressed) from the surface of spiro to the interface between spiro/perovskite.


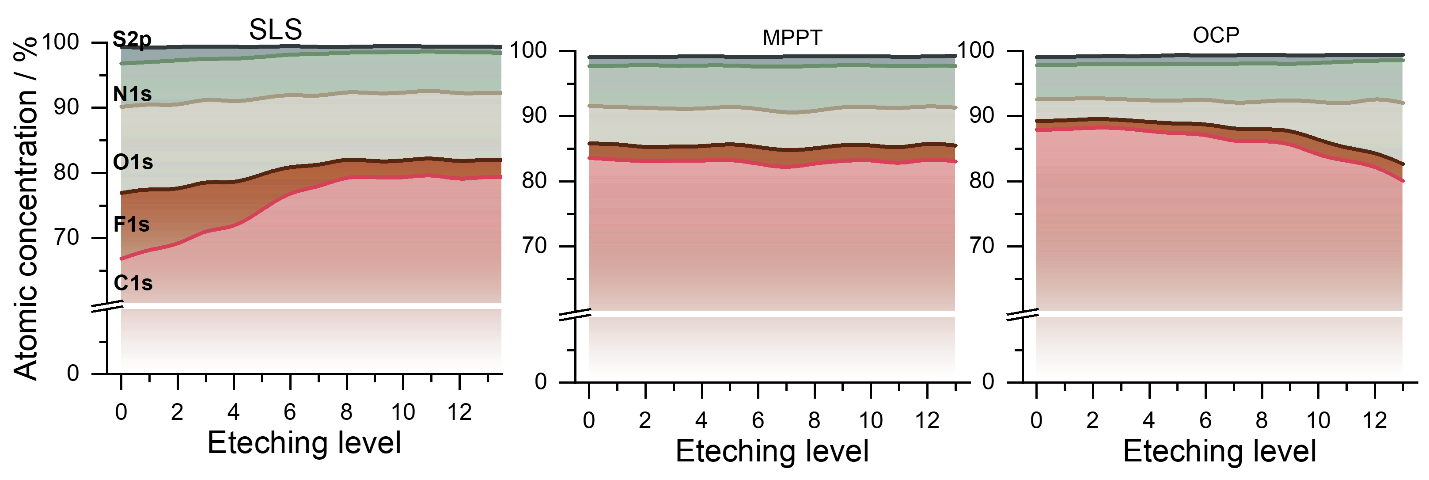


Figure S10: Elemental distribution from the surface to the interface of spiro/perovskite determined from the C-SDP in all the aged conditions.


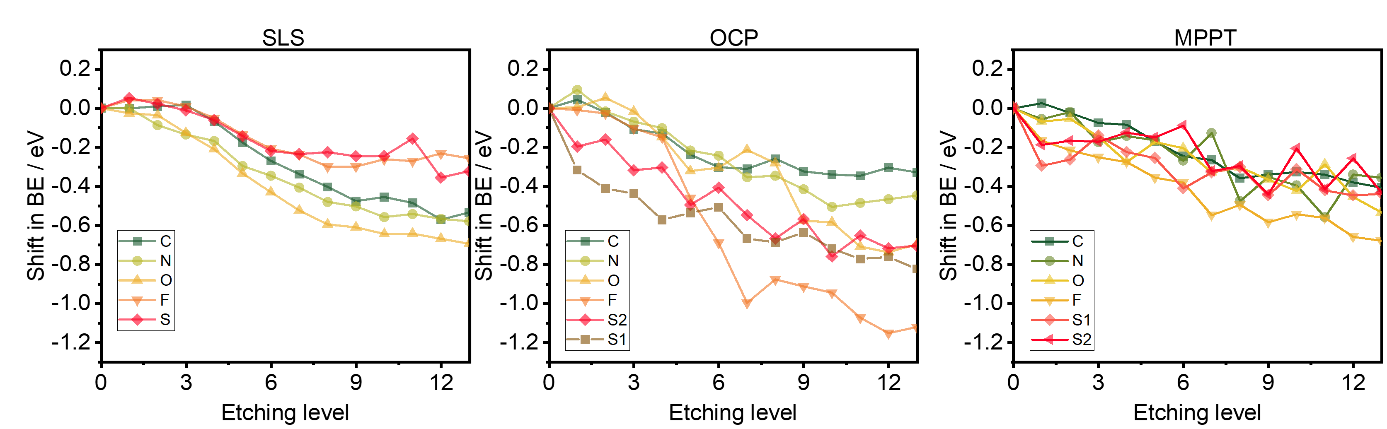


Figure S11. Relative shift in BE of various C1s, N1s, O1s, F1s, and S2p core levels in doped spiro in SLS, MPPT, and OCP-stressed devices from the spiro surface towards the spiro/perovskite interface.


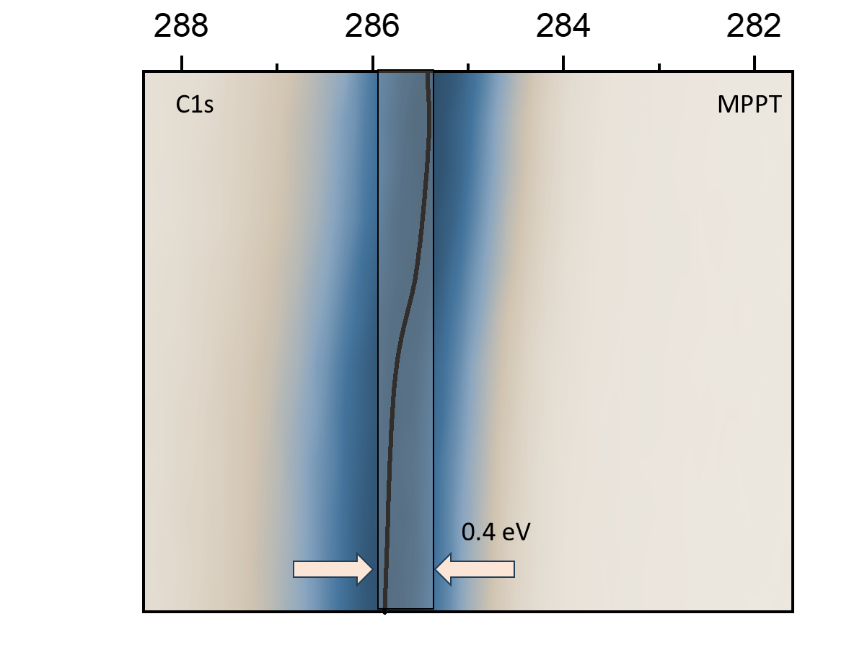


Figure S12: C1s spectral map from surface towards the interface of spiro/perovskite showing the direct band bending in the MPPT-stressed device.
